# Elevated temperature increases reproductive investment in less preferred mates in the invasive European corn borer moth

**DOI:** 10.1002/ece3.7972

**Published:** 2021-08-04

**Authors:** Arielle N. Enos, Genevieve M. Kozak

**Affiliations:** ^1^ Department of Biology University of Massachusetts‐Dartmouth Dartmouth Massachusetts USA

**Keywords:** climate change, Lepidoptera, mate choice, plasticity, sexual selection

## Abstract

Rapidly changing environments may weaken sexual selection and lead to indiscriminate mating by interfering with the reception of mating signals or by increasing the costs associated with mate choice. If temperature alters sexual selection, it may impact population response and adaptation to climate change. Here, we examine how differences in temperature of the mating environment influence reproductive investment in the European corn borer moth (*Ostrinia nubilalis*). Mate preference in this species is known to be related to pheromone usage, with assortative mating occurring between genetically distinct E and Z strains that differ in the composition of female and male pheromones. We compared egg production within and between corn borer lines derived from four different populations that vary in pheromone composition and other traits. Pairs of adults were placed in a mating environment that matched the pupal environment (ambient temperature) or at elevated temperature (5°C above the pupal environment). At ambient temperature, we found that within‐line pairs produced eggs sooner and produced more egg clusters than between‐line pairs. However, at elevated temperature, between‐line pairs produced the same number of egg clusters as within‐line pairs. These results suggest that elevated temperature increased investment in matings with typically less preferred, between‐line mates. This increased investment could result in changes in gene flow among corn borer populations in warming environments.

## Graphical abstract

We compared egg production within and between corn borer lines derived from populations that differ in pheromone similarity. Our results suggest that elevated temperature increases investment among populations. At elevated temperature (5°C above the pupal environment), between‐line pairs produced the same amount of egg clusters as within‐line pairs unlike at ambient temperatures (matching the pupal environment).

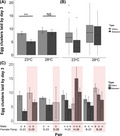

## 1 INTRODUCTION

Sexual selection and mate choice decision rules typically lead individuals to reject or invest minimally in a less preferred mate, in order to save resources for a highly preferred one (Anderson, 1994; Rosenthal, 2013, 2017). Environmental change may alter the availability of potential mates or increase the cost of expressing preference, weakening sexual selection (Botero & Rubenstein, 2012; Candolin & Wong, 2012; Chunco, 2014; Gillespie et al., 2014; Larson et al., 2019; Martin & Lopez, 2013; Rosenthal, 2013; Sih et al., 2011). In changed or stressful environments, there may also be increased costs associated with mate searching or increased probability of dying before completing reproduction, so it may be adaptive for animals to accept lower quality or less preferred mates, including mates from another species (Bateson & Healy, 2005; Edomwande & Barbosa, 2020; Forsgren, 1992; Pfennig, 2007; Real, 1990). Thus, environmentally based plasticity in mate choice can influence the degree of sexual selection, assortative mating, and hybridization rates among populations and species (Coomes et al., 2019; Rosenthal, 2013).

There is mounting evidence that increases in temperature interfere with the production or reception of mating signals and alter preference functions, shifting precopulatory mate choice (Boullis et al., 2016; Coomes et al., 2019; García‐Roa et al., 2020; Larson et al., 2019). A recent meta‐analysis found support for temperature effects on direct and indirect measures of sexual selection (García‐Roa et al., 2020). However, temperature effects appear to be variable among species and precopulatory sexual selection has been observed to increase, decrease, or remain unchanged. This variability in the direction of effects remains even when considering studies within a single animal group, such as insects, which are ectotherms and likely to be sensitive to changes in environmental temperature. For example, in *Osmia* Mason bees, chemical pheromone profiles of antennae change at elevated temperatures and decrease the attractiveness of males (Conrad et al., 2017). In several species of Lepidoptera, temperatures above 26°C reduce the specificity of male flight response to species‐specific female pheromone (Linn et al., 1988). While male song changes with temperature in *Drosophila montana*, female preferences do not shift accordingly (Ritchie et al., 2001). However, elevated temperatures do not always weaken sexual selection if temperature coupling occurs (Doherty, 1985). For example, in *Enchenopa* treehoppers, male vibrational courtship signals and female preferences change with temperature in the same direction, leading to consistency in sexual selection across temperatures (Jocson et al., 2019).

The majority of previous studies on sexual selection and temperature have focused on precopulatory mate choice, but there is also evidence that temperature alters postcopulatory interactions between males and females. In *Tribolium* beetles, females at high temperatures experience increased benefits to mating more than once (Grazer & Martin, 2012). Temperature alters the degree of sexual conflict in *Drosophila melanogaster* (Garci‐Roa et al., 2019). In *Lasioderma* cigarette beetles, comparison of effects of temperature on pre‐ and postcopulatory sexual selection found that temperature primarily altered postcopulatory sexual selection, increasing first male paternity (Suzaki et al., 2018). Cryptic female choice and investment in more attractive mates compared with typically less attractive mates may also be altered by temperature; however, few studies have investigated this.

Given this variability in the response of sexual selection and reproductive investment to increased temperature, we currently lack the ability to predict how many insect species will respond to changing climates, particularly those species where sexual selection and assortative mating maintain genetic differentiation among populations or strains that differ in host plant or pheromone usage. Predictions of response to climate change in insects typically focus on how environmental shifts will allow range expansion or increase metabolic rates (Deutsch et al., 2018), with little recognition for changes in reproduction that may occur through temperature‐induced plasticity. Recent work with *Drosophila* species suggests extinction rates under experimental conditions are related to male fertility limits rather than organismal survival limits (van Heerwaarden & Sgro, 2021). This gap in knowledge of how reproduction is affected by temperature is especially troubling for invasive species, which are predicted to have greater plasticity (Kelly, 2019), a greater aptitude for response to climate change (Bellard et al., 2013), and for which admixture among different source populations in the introduced range often contributes to adaptation and success (Ruis & Darling, 2014).

**FIGURE 1:**
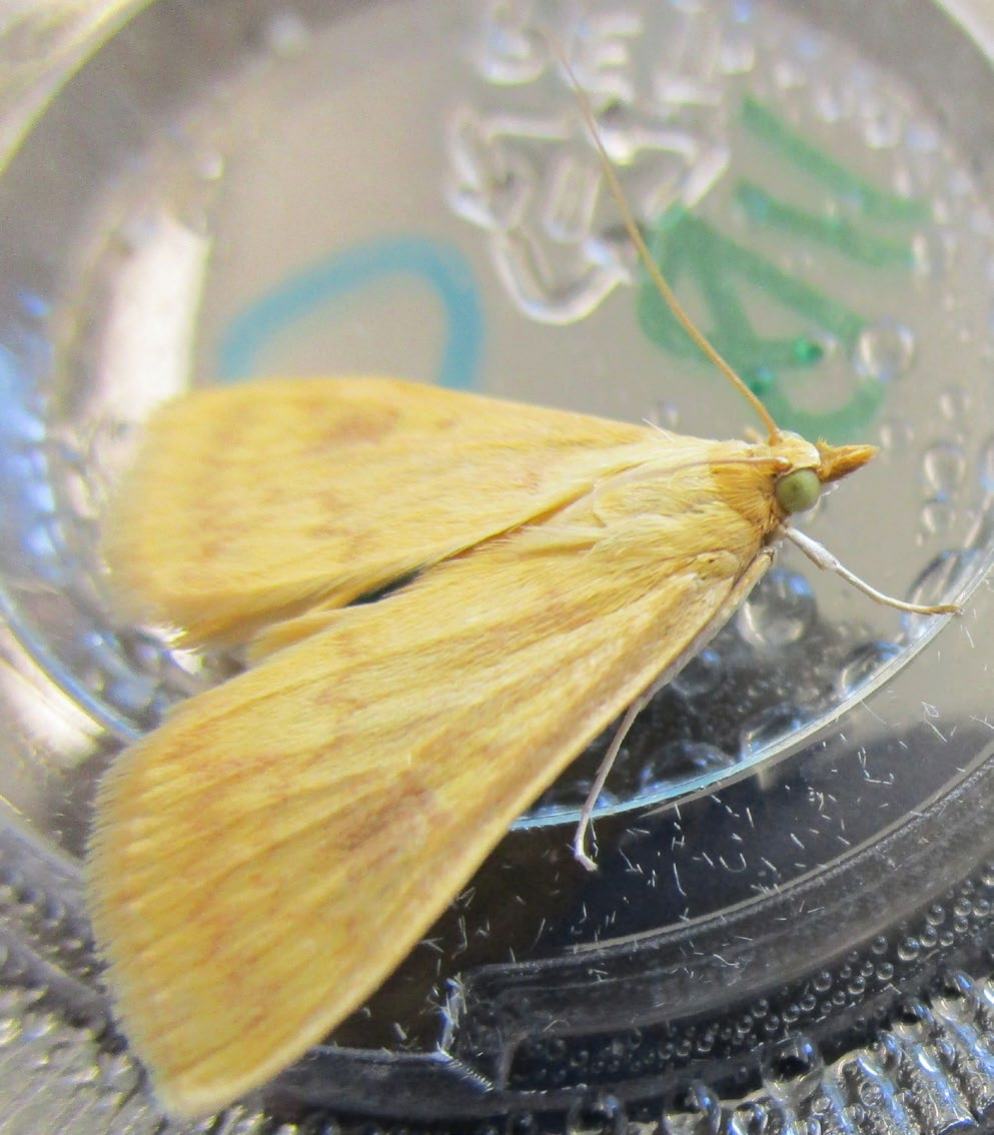
European corn borer female

In this study, we examine the effects of temperature on investment in reproduction at different temperatures in the invasive European corn borer moth (*Ostrinia nubilalis*; Figure 1). Corn borers are an agricultural pest originally introduced to North America from multiple locations in Europe in the early 1900s (Coates et al., 2018). This species has two strains that have diverged in chemical pheromone communication and have different preferred ratios of chemical isomers of 11‐tetradecenyl acetate in the female pheromone blend (Linn et al., 1997; Roelofs et al., 1985). In the E strain, females produce a 99:1 ratio of E to Z isomers, while Z strain females produce a 3:97 E:Z ratio. This divergence in long‐range pheromone leads to assortative mating by pheromone type and limits mating between strains, as males only respond and fly to their strain‐specific pheromone blend (Roelofs et al. 1987; Linn et al., 1997). In North America, sweet corn (*Zea mays*) is the primary host plant for both pheromone strains (Coates et al., 2019; O’Rourke et al., 2010). Previous work indicates that the E strain has increased cold tolerance compared with the Z strain (Wadsworth et al., 2020); however, differences in heat tolerance are unknown. Close‐range mating interactions also contribute to precopulatory mate choice and assortative mating among E and Z strains. Only 19% of E‐Z pairs mate after one night when they are placed together (eliminating the effect of male long‐distance orientation), compared to 61% of within‐strain pairs (Dopman et al., 2010).

In North American populations of corn borers, genomic differentiation is linked to pheromone strain identity rather than geographic distance or host plant usage (Coates et al., 2019). The analyses of population genetic structure have identified E and Z clades, as well as a third group of hybrid/admixed individuals present at sympatric sites (where both strains occur at high frequency: Coates et al., 2019; H. D. Kunerth, G. M. Kozak, E. B. Dopman, unpublished data). Genetic loci for female pheromone production and male response to pheromone have been identified, are located on different chromosomes, and are maintained in linkage disequilibrium by assortative mating (Dopman et al., 2004; Unbehend et al., 2021). Genetic differences among corn borer populations are also related to a large region on the sex chromosome that shows repressed recombination between E and Z strains (Wadsworth et al. 2015; Kozak et al., 2017). Populations appear to vary in the degree to which this region is differentiated due to differences in seasonal mating time and voltinism among strains (Kozak et al., 2017, 2019). Nearly all matings between pheromone strains produce viable offspring and any reductions in fitness interstrain hybrid experience are related to failure to attract mates (Dopman et al., 2010; Liebherr & Roelofs, 1975). E and Z strain males have low response to intermediate pheromones that hybrid females produce (65:35 E‐Z), although hybrid males respond to most types of female pheromone (Roelofs et al. 1987; Linn et al., 1997). Previous work suggests that when individuals are paired in the laboratory, hybrid females obtain matings with E and Z males at an intermediate rate, suggesting that pure strain males discriminate against them (Liebherr & Roelofs, 1975; Pélozuelo et al., 2007).

Both males and females may be selective of mates and invest differentially in mates in the European corn borer. European corn borer females typically mate 1–3 times over their approximately 14‐day adult life, with the majority of females tending to mate once and males typically mating over 3 times (Elliott, 1977; Fadamiro & Baker, 1999; Hinton & Andow, 2003; Lee & Spence, 1987). Precopulatory female choice is mainly expressed in corn borers by a female moving away to reject a male or limiting access to her abdomen (Dopman et al., 2010; Farrell & Andow, 2017). Males also exhibit choice by choosing not to court females, and they do not appear to coerce females. Behavioral observations suggest decreases in mating between strains are due to both reduction in male courtship initiation and female rejection of male courtship attempts when they do occur (Dopman et al., 2010); however, the specific mating traits that contribute to close‐range rejection have not yet been conclusively identified. When a European corn borer female accepts a male as a mate, they copulate and the male transfers a nutrient‐rich spermatophore. Possible postcopulatory interactions that affect the number of eggs laid include male adjustment of spermatophore (size or content), female remating rate, and female investment in egg laying after a mating (Fadamiro & Baker, 1999; Royer & McNeil, 1993).

To determine whether temperature impacts reproduction among corn borer populations, we compared rates of egg production over the first three days after pairing males and females at ambient temperature (23°C) and elevated temperature (28°C). We paired females and males within and between four lines originating from different populations that had been maintained in the laboratory for many generations (Table 1). Based on previous work suggesting mating is related to pheromonal and genetic differences among populations, we predicted pairs within lines would mate more frequently and produce more eggs than pairs between lines. We analyzed whether or not any egg clusters were produced over a given time period as a proxy of mating occurring (unmated females do not lay eggs in clusters of more than 5 eggs: Leahy & Andow, 1994). We used the number of egg clusters laid during a given time period as a measure of reproductive investment of male or female resources to a given mate. If investment in eggs remained the same at elevated temperatures, it would suggest that temperature has little overall effect on reproduction. Alternatively, we might see changes at elevated temperatures if the costs and benefits of investment in a given mating are altered by temperature. Differences in the effects of temperature on within‐ compared to between‐line mating pairs may indicate whether these temperature effects are general or specific to potentially less preferred mates. Whether temperature‐induced changes occurred in the production of eggs or overall investment in eggs would give insight into potential mechanisms.

**TABLE 1:** Population site information. Temperature data from NOAA National Centers for Environmental Information (2020)

Line	Pheromone strains present	Peak mating time	Site location	Pairs 23°C	Pairs 28°C	Average maximum July temperature (1978–2018), °C
BV	100% Z	Univoltine; July	Bouckville, Madison Co., NY	14	14	26.1
DV	89% E	Bivoltine; June/August	Dover, Norfolk Co., MA	15	87	28.4
HR	64% E 36% Z	Bivoltine; June/August	Hurley, Ulster Co., NY	11	9	27.1
GE	100% E	Bivoltine; June/August	Geneva, NY Ontario Co., NY	11	13	26.9

## 2 METHODS

For mating trials, we used lines derived from four different field populations. One of our laboratory‐bred lines was derived from a univoltine Z‐strain population in Bouckville–Madison, NY (100% Z strain, Z clade: Glover et al., 1991; Dopman et al., 2005). A second line was derived from a bivoltine E‐strain population in Geneva, NY (100% E strain, though this site has both E and Z present; Dopman et al., 2005; Wadsworth et al., 2020). A third line was from Dover, MA, a site which is dominated by bivoltine E strain. At Dover, pheromone traps indicate 96%–81% of trapped individuals are E‐pheromone preferring and genetic analysis suggests 89% of individuals are genetically E clade (H. D. Kunerth, G. M. Kozak, E. B. Dopman, unpublished data). A fourth line was derived from a population in Hurley, NY, where E and Z pheromone types co‐occur, and in the field, 64% of trapped individuals are E‐strain preferring (Zuefle, 2018). Extensive genetic data are not yet available for Hurley, but similar sites with equivalent ratios of E and Z pheromone usage have a large proportion of genetically admixed individuals (Coates et al., 2019; H. D. Kunerth, G. M. Kozak, E. B. Dopman, unpublished data) and heterozygous genotypes at the female pheromone locus were documented among a small sample of Hurley founders (3 out of 4 genotyped).

All lines had been mass‐reared in the laboratory for multiple generations (>15 generations) at 23–26°C degrees (depending on the generation; most were reared at constant 23°C or constant 26°C; see Appendix Table S1) and fed an artificial corn‐based diet (Southland Products, Lake Village, AK). The Hurley line (HR) had been established in April 2018 from diapausing larvae collected from corn stubble at an organic farm in Hurley, NY (60 individuals establishing). The Dover colony (DV) was established from individuals collected as direct‐developing caterpillars from corn stalks in July 2018 from an organic farm in Dover, MA (60 individuals establishing). The Bouckville line (BV/UZ) and Geneva line (GE/BE) were obtained from colonies maintained at Tufts University that originated at the New York State Geneva Agricultural Station (Wadsworth et al., 2013). Each generation after founding, mating occurred in cages where 100–200 mating individuals per generation were placed and allowed to mate freely for 7–10 days after which 300–600 egg clusters were randomly sampled from all of those produced for the next generation.

Ambient and elevated temperature exposures were selected to differ by 5°C because an increase in global temperature of 5°C by 2,100 is the upper limit of the more extreme global change models (IPCC, 2014). In addition, 28°C was the highest average monthly maximum temperature observed at any of our population collection sites (Table 1; NOAA National Centers for Environmental Information, 2020). The ambient temperature (23°C) was the maximum temperature estimated to be experienced by the pupal stage of E strain in Geneva, NY (Wadsworth et al., 2020), and a temperature experienced by our lines over many generations in the laboratory.

When individuals reached the pupal stage, they were isolated in 29.6‐ml cups and held at constant 23°C for at least 7 days prior to eclosion to adulthood (>50% of the pupal duration as pupation‐eclosion time is typically 10–13 days: Kozak et al., 2019). Pupae were held in a temperature‐controlled I‐36VL incubator (Percival Scientific, Perry, IO) at UMass‐Dartmouth, set to a 16:8 hr (L/D) cycle, and maintained around 60% humidity. Temperature and humidity were verified and tracked with an AcuRite digital monitor (Lake Geneva, WI). Pupae were checked daily, and date of eclosion was recorded. Adults were then held at 23°C from eclosion until mating trial placement (average 1.69 days held as adults). In our experiment, we did not allow for adults to acclimate to elevated temperatures, mimicking adults experiencing a heat wave during reproduction. We hypothesized that this adult temperature treatment might influence females more than males because egg and sperm development are offset developmentally in corn borers, with egg development primarily in the first few days occurring after pupal eclosion (Miller, 1988) and sperm development occurring during the final pupal stages (Chaudhury & Raun, 1966). Isolating pupae ensured all adults were unmated at the time of pairing. Thus, while temperatures experienced by eggs and larvae varied (23°C, 26°C, and for a small number, 28°C for <7 days), pupal and adult temperatures were controlled (see Appendix Table S1 for details on larval temperature).

When mating pairs were set up using adult males and females, individuals were transferred to elevated temperatures (28°C) or kept at ambient temperatures (23°C). Given prior evidence that Z strain females from European populations prefer older males and male pheromone may be an honest signal of age (Lassance & Löfstedt, 2009), we kept track of male and female age. The first day of eclosion was recorded as age “Day 0.” Mating pairs were set up from the available adults on any given day, splitting individuals of different ages across temperature treatments and pair types as evenly as possible. Mating pairs were placed in 236‐mL paper cups with wax paper on the side for egg laying, a mesh top secured by a rubber band, and free access to tap water via dental cotton threaded through the base that sat in a water reservoir below. For the elevated temperature trials, mating pairs were transferred to a second Percival Incubator maintained at 28°C and 60% humidity on the same 16:8 hr (L/D) cycle (housed in the same room as the 23°C incubator). All mating trials were run between August 2019 and December 2020.

We measured 197 pairs total: 95 within line (23°C = 53, 28°C = 42) and 102 between line (23°C = 54, 28°C = 48; Table 1; Table 2). Pairs were set up in the late afternoon (2‐6p.m.) and then checked daily for eggs in the early afternoon (between 11 a.m. and 3 p.m.) on consecutive days. We used the presence of egg clusters as an indicator of when mating had occurred. Mated *Ostrinia* females begin laying eggs in clusters of 15–20 eggs one day after mating (Royer & McNeil, 1993), while unmated females occasionally lay single eggs or clusters of less than five eggs (Leahy & Andow, 1994). We only recorded egg clusters containing more than five eggs. We counted the number of egg clusters daily for six days after pairing. Latency was measured as the day on which the first egg clusters were observed. If pairs had produced no egg clusters by the 6th day, we recorded a latency of six for the pair. Due to the small size of *Ostrinia* eggs, counts of egg clusters are more accurate than counts of egg numbers. After the trial, for a subset of the pairs (*N* = 76), we counted the total number of eggs and calculated the number of eggs per cluster. This value did not differ between females from different lines or temperatures (all *F*_3,62_ < 1.21, *p* > 0.31), with average eggs per cluster = 15.42 ± 0.58 SE. If pigmented eyespots were observed in eggs (a developmental stage that occurs 24hrs before hatching: Royer & McNeil, 1993), the number of clusters with eyespots was counted and we froze the pair (this could occur on Days 4–6 depending on when the first egg clusters appeared).

**TABLE 2:** Between population pairs. Number of pairs for each between population cross listed

Cross (Female × Male)	Pairs 23°C	Pairs 28°C	Total Pairs 23°C	Total Pairs 28°C	Distance between sites (km)
HR × DV	9	7	**15**	**8**	297
DV × HR	6	1			
HR × BV	10	9	**15**	**16**	210
BV × HR	5	7			
GE × BV	11	10	**24**	**24**	141
BV × GE	13	14			

Totals for each population pair (all female‐male combinations) shown in bold.

All statistical analyses were done in R v.3.5 (R Core Team, 2018). To determine whether mate identity or temperature influences how quickly mating occurs, we tested for differences in latency (in days) to lay eggs in a generalized linear model with a Poisson distribution. This latency analysis found a median latency of 3 days, so we used number of egg clusters present on the third day after pairing as a metric of reproductive investment. This is similar to previous studies that measured mating success in corn borers 3–4 days after pairing (Liebherr & Roelofs, 1975; Pélozuelo et al., 2007) and minimizes any effects of acclimation to elevated temperature in individuals that delay mating. We tested if between‐line crosses involving the admixed/hybrid Hurley population were different than between‐line crosses involving “pure” E and Z strains, but found no difference in any mating measures (all *Z* < 0.41, *p* > 0.68), so we analyzed “pair type” as simply within or between line. To account for age, we used the age class on the day of pairing for both females and males (Females: Day 0 *N* = 20, Day 1 *N* = 93, Day 2 *N* = 50, Older *N* = 34; Males: Day 0 *N* = 20, Day 1 *N* = 84, Day 2 *N* = 60, Older *N* = 33). To determine whether mate identity and temperature alter the number of egg clusters produced by the third day after pairing, we analyzed the number of egg clusters on the third day using a zero‐inflated negative binomial regression in the pscl package in R (Jackman, 2020; Zeilis et al., 2008). This model examined how the probability of producing eggs and the number of egg clusters produced was influenced by pair type (within/between line), temperature (ambient/elevated), line of origin of the female (HR, DV, BV, GE), female or male age class at the time of pairing (Day 0, Day 1, Day 2, Older), and interactions between temperature and all factors. Nonsignificant interactions were removed to simplify the models (all *p* > 0.12). We calculated likelihood ratio chi‐square statistics for factors in the car package (Fox & Weisberg, 2019) and performed Tukey‐corrected pairwise comparisons using emmeans (Lenth, 2019). To further analyze and visualize effects, we used several other R packages: dplyr (Wickham et al., 2018), ggplot2 (Wickham, 2016), MASS (Venables & Ripley, 2002), plyr (Wickham, 2011), survival (Therneau, 2020), and survminer (Kassambara et al., 2019). The standard error (SE) for the proportion producing eggs was calculated using the equation SE = $\sqrt{p\left(1‐p\right)/n}$ (Havel et al., 2019). To verify that our results were not the result of larval temperatures, we also analyzed the subset of crosses that experienced constant 23°C during larval development (*N* = 126, within line *N* = 68, between line *N* = 58).

## 3 RESULTS

**FIGURE 2:**
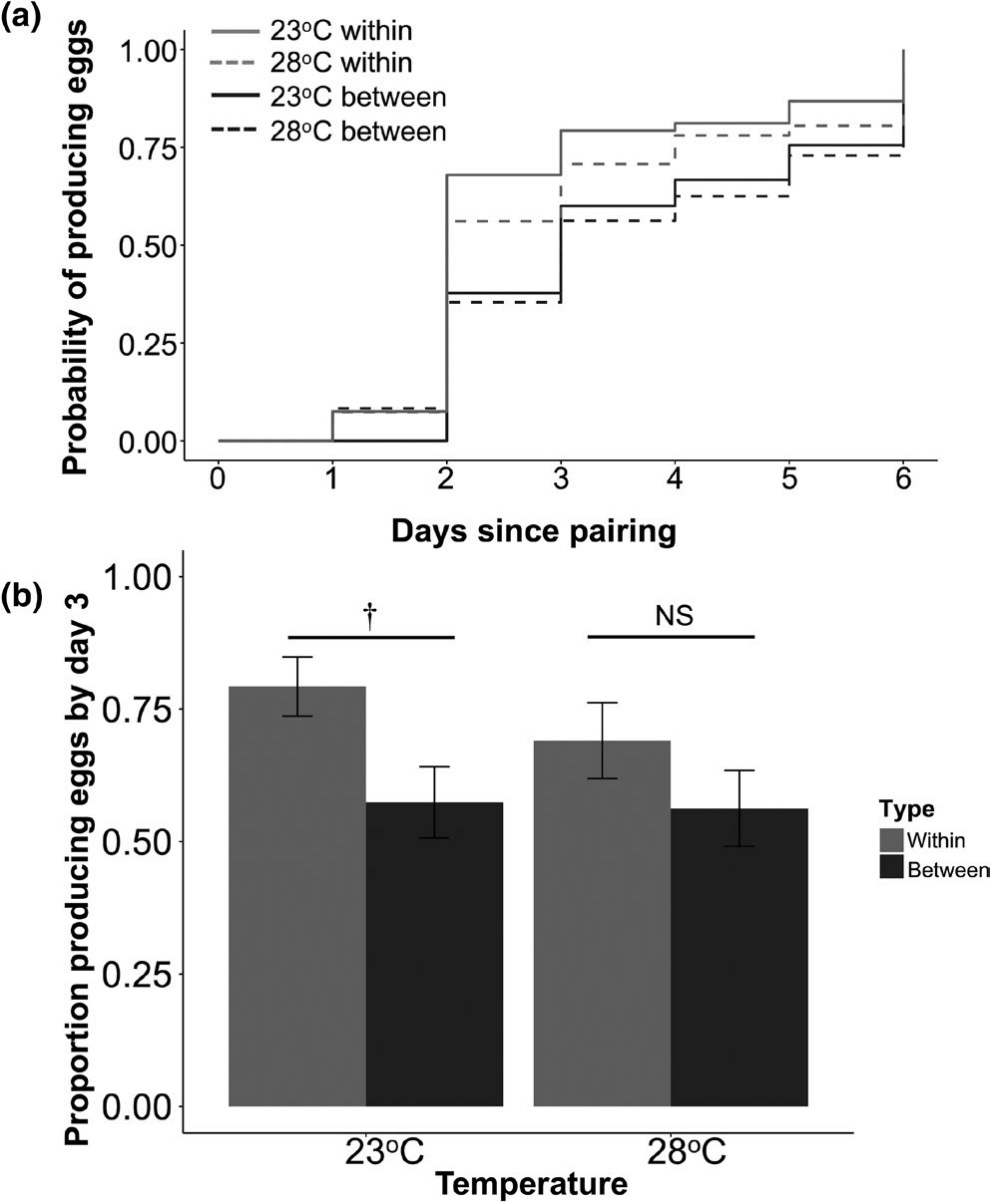
Egg production of between‐ and within‐line pairs at ambient (23°C) and elevated (28°C) temperature. (a) Latency to produce eggs with the cumulative proportion of pairs that had produced eggs plotted on each day; solid lines = ambient temperature, dashed lines = elevated temperature. (b) Proportion producing any eggs by the third day after pairing. Light gray = within‐line pairs, dark gray = between‐line pairs. Tukey‐corrected post hoc *p*‐values shown above: **p < 0.01; *0.01 < *p *< 0.05; †0.05 < *p* < 0.10; NS = nonsignificant

Analysis of latency to produce eggs found that between‐line pairs took longer to lay eggs than pairs from the same line, independent of temperature (difference: 0.69 ± 0.26 days; $\chi_{1}^{2}$ = 5.39, *p* =0.02; Table 3; Figure 2a). Mean latency for within‐line pairs was 3 days (23°C = 2.77, 28°C = 3.07), while latency for between‐line pairs was closer to 4 (23°C = 3.56, 28°C = 3.64). Females from the DV line tended to have longer latencies to produce eggs compared with the other three lines (DV‐GE: *p* = 0.01, Tukey‐adjusted *p* = 0.07; DV‐BV: *p* = 0.002, Tukey‐adjusted *p* = 0.011, DV‐HR: *p* = 0.04, Tukey‐adjusted *p* = 0.18).

**TABLE 3:** Analysis of latency to produce eggs

Factor	*df*	*χ* ^2^	*p*
Type (within, between)	1	5.38	0.02
Temperature (23,28)	1	1.88	0.17
Female line	3	9.81	0.02
Male age	3	2.00	0.57
Female age	3	9.39	0.024

Pair type had a significant effect on whether or not eggs were laid by the third day, which was also independent of temperature ($\chi_{1}^{2}$ = 7.08, *p* = 0.007). A higher percentage of within‐line pairs had eggs by the third day after pairing (within 23°C = 0.79 ± 0.06; 28°C = 0.69 ± 0.07) than between‐line pairs (between 23°C = 0.57 ± 0.07, 28°C = 0.56 ± 0.07; Table 4; Figure 2b). Although the interaction between temperature and pair type was nonsignificant and removed from our model (*p* = 0.48), differences among the proportion producing eggs for within‐ versus between‐line pairs were more pronounced at ambient temperature (within–between = 0.22 ± 0.10, *p* = 0.019, Tukey‐adjusted *p* = 0.09) than at elevated temperature (within–between = 0.15 ± 0.10, *p* = 0.14, Tukey‐adjusted *p* = 0.44). Furthermore, female line of origin did have an effect on probability of producing eggs, with DV females being less likely than BV and GE females to produce eggs by the third day (all Tukey‐adjusted *p* <.03; DV‐HR *p* = 0.23; Appendix Figure S1).

**TABLE 4:** Zero‐inflated negative binomial analysis of probability of producing egg clusters and number of egg clusters produced by Day 3

Factor	*df*	Probability of producing eggs		Number of egg clusters	
		*χ* ^2^	*p*	*χ* ^2^	*p*
Type (within, between)	1	7.08	0.008	0.24	0.62
Temperature (23,28)	1	2.50	0.11	8.87	0.003
Female line	3	12.20	0.007	1.55	0.67
Male age	3	1.44	0.69	1.62	0.66
Female age	3	6.56	0.09	3.48	0.32
Temperature: Type	1	NA	NA	4.44	0.035

**FIGURE 3:**
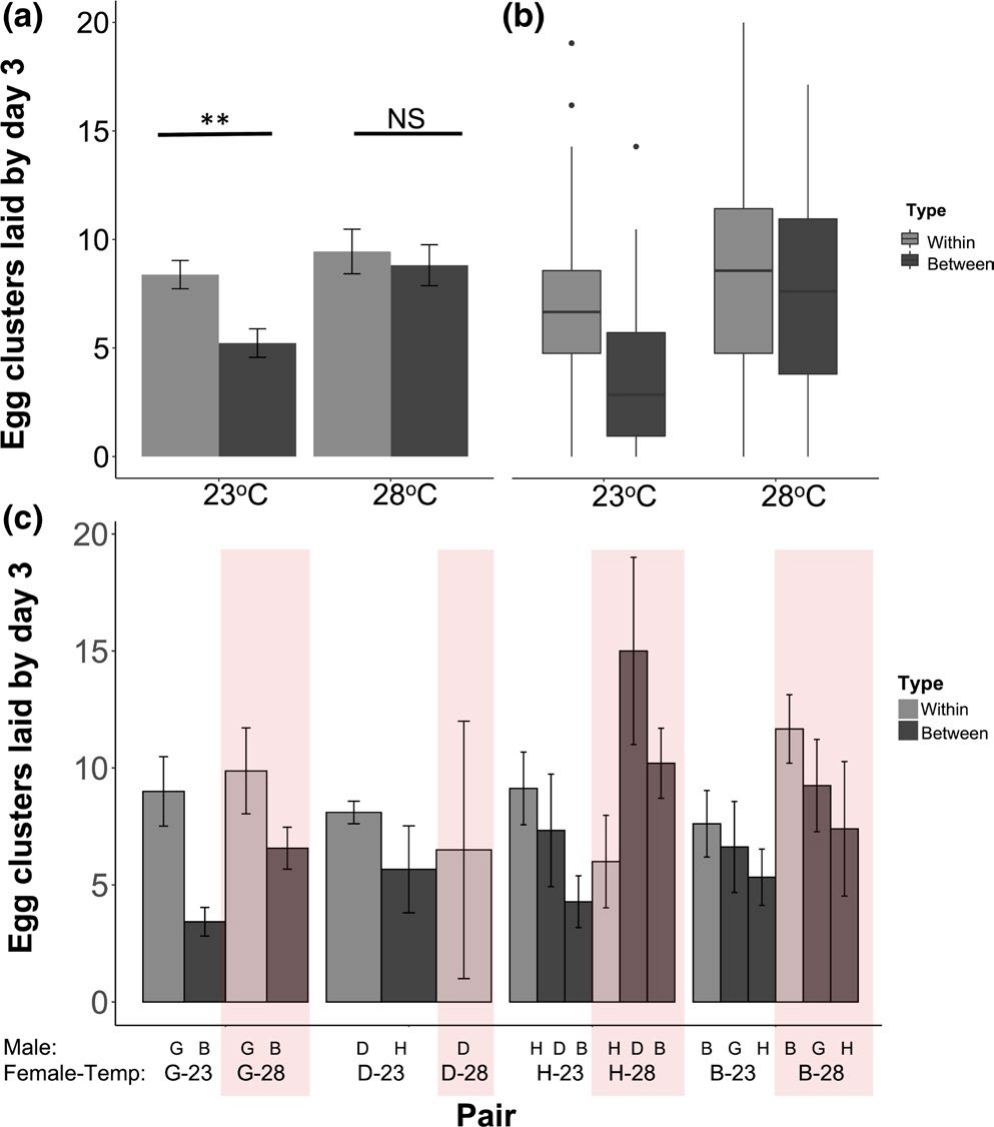
Egg clusters laid by between‐ and within‐line pairs at ambient (23°C) and elevated (28°C) temperature (excluding those that did not produce eggs). (a) Average number of egg clusters produced by the third day across all pairs, error bars represent standard error. Tukey‐corrected post hoc *p*‐values shown above: **p < 0.01; *0.01 < *p* < 0.05; NS = nonsignificant. (b) Boxplot of the number of egg clusters laid by the third day for those that did lay eggs (excluding those that did not produce eggs). (c) Egg clusters laid by day 3 by male line, female line, and temperature. Male line listed above, female line and temperature listed below. No DV female by HR male pairs produced eggs, so this group not shown on plot. 28°C pairs shaded in red. No interactions between female line and temperature were significant. G = GE/Geneva D = DV/Dover; H = HR/Hurley, B = BV/Bouckville. Sample sizes listed in Appendix Table S2. Light gray = within‐line pairs, dark gray = between‐line pairs

We found that the number of egg clusters laid by the third day varied based on pair type and temperature (interaction $\chi_{1}^{2}$ = 4.44, *p* = 0.035; Table 4). At ambient temperature, within‐line pairs laid more egg clusters than between‐line pairs (*Z* = 3.28, *p* = 0.001, Tukey‐adjusted *p* = 0.0057), but at elevated temperature, the number of egg clusters laid in both types of pairs was the same (*Z* = 0.52, *p* = 0.60, Tukey‐adjusted *p* = 0.95; Figure 3a, b). This pattern is due primarily to an increase in the number of egg clusters laid between lines at 23 and 28°C (*Z* = −2.94, *p* = 0.003, Tukey‐adjusted *p* = 0.017). The female's line of origin did not significantly influence the number of egg clusters laid (Table 4) and interactions between female line and other factors were not significant (all $\chi_{3}^{2}$ < 3.18, *p* > 0.36; Figure 3c). The average rate of egg production, even when accounting for those pairs that produce zero eggs is similar at elevated temperature between and within lines, is higher for within‐line pairs at ambient temperature (ambient within–between *Z* = 3.03, Tukey‐adjusted *p* = 0.013; elevated within–between: *Z* = 1.01, Tukey‐adjusted *p* = 0.75). Pigmented eyespots (viable embryos) were observed at 28°C in roughly equal frequencies among pair types: 76% of within‐line pairs and 69% of between‐line pairs (*N* = 58; Fisher's exact test, *p* = 0.77). Due to slower development at 23°C, we did not observe these pairs long enough to see pigmented eyespots.

**FIGURE 4:**
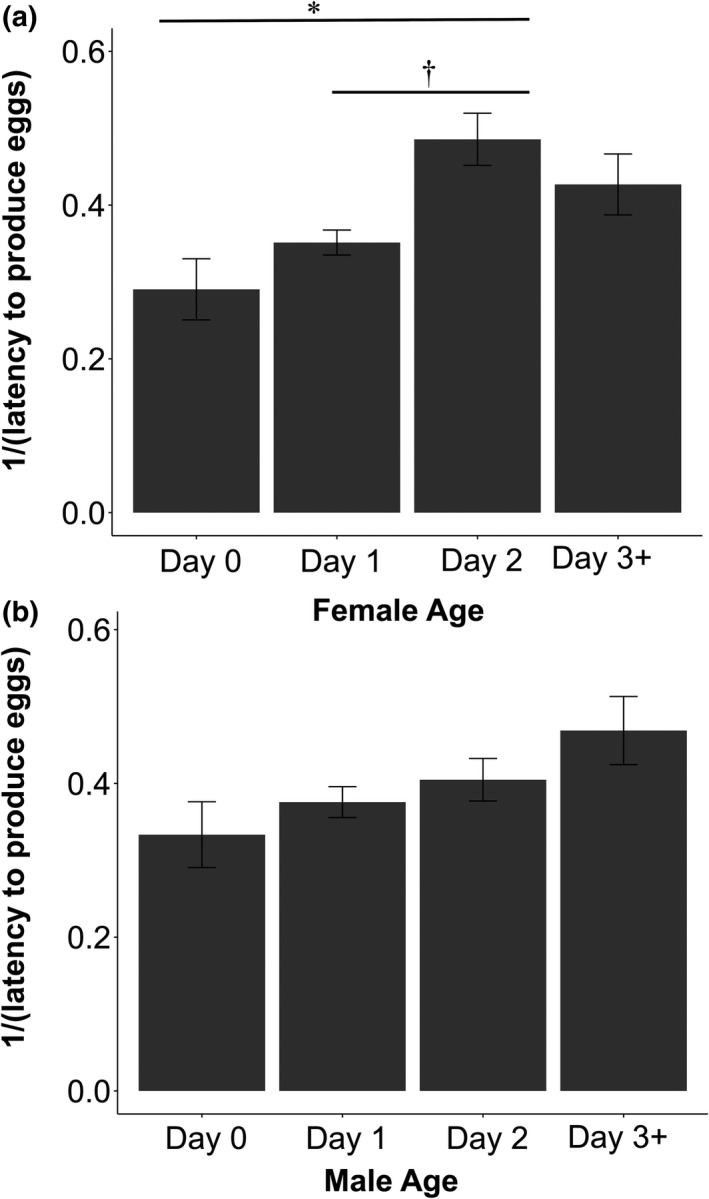
Inverse of latency to produce eggs and age at the time of pairing. (a) 1/(Latency to produce eggs) based on female age. Two‐day‐old females tended to produce eggs slightly faster than younger females. Bars represent standard error. (b) 1/(Latency to produce eggs) based on male age. Tukey‐corrected post hoc *p*‐values shown above: *0.01 < *p* < 0.05; †0.05 < *p* < 0.10. (Females: Day 0 *N* = 20, Day 1 *N* = 93, Day 2 *N* = 50, Older *N* = 34; Males: Day 0 *N* = 20, Day 1 *N* = 84, Day 2 *N* = 60, Older *N* = 33)

Female age influenced only latency to produce eggs with two‐day‐old females tending to lay eggs faster than younger females (Day 2 versus. Day 0: *Z* = 2.48, *p* = 0.008, Tukey *p* = 0.037; Day 2 versus. Day 1: *Z* = 2.47, *p* = 0.01, Tukey *p* = 0.06; Table 3; Figure 4a). These effects did not vary with temperature (all interactions *p* > 0.52 and removed from the model). Male age did not have any effects on egg production (all *p* > 0.50; Tables 3, 4; Figure 4b).

Restricting our analysis to the subset of crosses that experienced constant larval temperature of 23°C during development (*N* = 126 pairs, 79 of which produced egg clusters by Day 3), we still found that the number of egg clusters laid by the third day increased only in between‐line crosses at high temperatures (interaction $\chi_{1}^{2}$ = 10.94, *p* < 0.001; within–between ambient *Z* = 2.88, Tukey‐adjusted *p* = 0.02; within–between elevated temperature *Z* = −1.82, Tukey‐adjusted *p* = 0.27; Appendix Figure S2).

## 4 DISCUSSION

We found that elevated temperature in the mating environment increased investment in reproduction in the European corn borer but only when mating with typically less preferred, between‐line mates. At ambient temperatures, between‐line pairs took longer to produce eggs and produced fewer egg clusters overall. However, when temperature was elevated, females laid a similar number of egg clusters with mates within and between lines, resulting in a weakening of preferential investment at elevated temperatures.

The change we found in reproductive investment at elevated temperatures may be an adaptive response to temperature in order to limit the costs of reproduction, by investing more in reproduction earlier in life to offset higher mortality or shorter life spans at high temperatures. In many insects, including corn borers, there is a trade‐off between fecundity early in life and female longevity, with females that lay more eggs early in oviposition having shorter life spans (Lee & Spence, 1987; Reznick, 1985). Our results are consistent with investment in reproduction being at its maximum for within‐line pairs, but at elevated temperatures, females may choose to invest more eggs in a mating with a less preferred mate rather than reserving resources or waiting for more highly preferred mate. This pattern may be indicative of shifts in postcopulatory female choice and usage of male sperm for fertilization. If increased costs at high temperature drive these temperature effects, then increases in female resource availability or condition may mitigate changes in investment. However, few studies on sexual selection and reproduction under environmental change have investigated the potential interaction between resource availability and environmental stressors such as temperature.

Determining what behavioral or physiological mechanisms may underlie the changes in reproduction we document and how these mechanisms vary among populations or sexes is necessary to understand the impact rising temperatures will have on corn borers and other invasive insects. Temperature has a number of physiological effects that can alter reproductive traits (reviewed in García‐Roa et al., 2020). Sperm and egg performance are altered at increased temperature in *Tribolium* beetles, although documented plastic changes that improve performance only occur in individuals experiencing high temperatures at early life stages, rather than as adults (Vasudeva et al., 2019). Females may develop their eggs faster at higher temperatures, as egg development in corn borers occurs mostly during the first day after eclosion (Miller, 1988). Male corn borer moths might also alter the contents of the spermatophore that they provide to less preferred mates or at different temperatures, because males of a closely related moth species (*Ostrinia scapulalis*) are known to adjust the size and protein content of the spermatophore based on the size of the female (Win et al., 2013). Another possibility is that our results are driven by changes in multiple mating rate or a female's intermating interval. Female multiple mating rates increase with temperature in many insects, including Gryllidae crickets (Kindle et al., 2009), *Tribolium* beetles (Grazer & Martin, 2012), and *Lasioderma* cigarette beetles (Suzaki et al., 2018). All of these potential mechanisms by which temperature impacts egg production with less preferred mates could be explored in future work, particularly by experiments independently manipulating late larval temperature or pupal temperature.

Predicting population persistence in changing environments requires knowledge of how sexual selection will change based on the environment (Fox et al., 2019; Kelly, 2019). Condition‐dependent sexual selection is predicted to improve adaptation to changing environments in large populations (Martínez‐Ruiz & Knell, 2017); however, if the strength or direction of sexual selection changes with the environment, sexual selection could hinder adaptation to changing climates. Mate choice changes dynamically in many species in changed environments, often weakening sexual selection (Candolin et al., 2007; Candolin & Wong, 2012). Our results suggest that rapidly warming environments may potentially promote gene flow among populations of invasive insects by increasing egg laying after mating with less preferred mates. Future work on how cryptic female choice or other changes to reproductive investment shift in warming environments is needed to fully understand the implications of this context‐dependent reproductive investment.

We did not directly test how differences between lines in pheromone composition led to the reduced investment between lines. Female pheromone was just one component that varies among the populations that we used to establish our lines, as they differ in seasonal timing and local temperature as well (see Table 1). Future work quantifying genetic and pheromonal differences among individuals would determine whether pheromonal attributes are the primary reason for reduced investment in between‐line mates. Potential traits that vary and could contribute to close‐range mating success are male pheromone, ultrasonic song, and circadian timing of courtship (Karparti et al., 2007; Lassance & Löfstedt, 2009; Levy et al., 2018; Liebherr & Roelofs, 1975; Takanashi et al., 2010). Gene flow among strains in wild populations of corn borers will also be dependent on the effects of temperature on other components of mating in corn borers. Breeding time (early vs. late dormancy emergence by temporal ecotypes) and male localization of females also contribute substantially to reduced mating rates among corn borer populations (Dopman et al., 2010). More work is needed to determine there are interacting effects of temperature on various components of mating in corn borers, but previous studies suggest it is likely that some of these other traits may also change with temperature. Elevated temperature has been found to reduce the difference among early and late emerging temporal ecotypes in corn borers (20°C: ecotype pupation difference = 48 days; 30°C: difference = 13 days) (Skopik & Takeda, 1987). In addition, in several other moth species, males become less specific in their response to female pheromone response as temperatures rise (Linn et al., 1988), suggesting corn borer males may as well. Multiple additive effects of temperature on different reproductive isolating barriers, combined with the changes in reproductive investment we document (if these are related to pheromone), could potentially lead to changes in gene flow among corn borer populations and hybridization among strains.

In this study, we used no‐choice tests in our experimental design and in the wild, males and females will likely have more opportunities to express both pre‐ and postcopulatory choices, even at elevated temperatures. Experimental testing of female mating behavior in a setting where females choose between different males (rather than either mating with a given male or not mating) would be informative for understanding the impact of elevated temperatures on reproductive investment and most importantly, on assortative mating itself. In addition, future work could use genetic data to estimate actual hybridization rates of European corn borer strains when males and females can freely interact in mesocosms at different temperatures, as our results suggest that all offspring remain viable at high temperatures because we found no reduction in the percentage of between‐line pairs with pigmented eyespots. Such future studies could fully quantify the impact of temperature on mating behavior and investment in the European corn borer.

We observed some variability among our different lines in the strength of temperature effects, although female line of origin was only significant for the probability of producing eggs by Day 3 and latency to lay eggs (Tables 3, 4, Appendix Figure S1). This may suggest that there is some genetic variability among lines in response or sensitivity to temperature, which could be explored in future work. The differences we found suggested that females from the BV line, which is a population that mates in the middle of the summer (univoltine), were less impacted by temperature than the DV line, which is bivoltine and mates twice (both earlier and later in the year). Previous studies found that the bivoltine GE line was more cold‐tolerant than the BV line (Wadsworth et al., 2020) and that temperature effects on fecundity may differ among populations (Lee & Spence, 1987). The BV line females also appeared to shift their investment in eggs less than females from other populations (Figure 3c), although female line was not significant in this analysis. It is possible that limited sample sizes for specific temperature treatments may have led to nonsignificance of interactions of female line, so further exploration of population differences in response of females and males is warranted. Populations often differ in the magnitude and direction of response to environment (commonly referred to as a genotype by environment or “GxE” interaction) (Fox et al., 2019), and exploring these differences in response of reproduction to temperature is another avenue for future work in order to understand how corn borer populations might respond to global change.

We think it is unlikely that changes in the genetic composition of our DV or HR lines over time produced differences between 23 and 28°C treatments for several reasons. First, the majority of the data collected from the HR and DV populations were collected in January–March 2020, which represents only two corn borer generations. In fact, 91% of the BV and HR pairs had data collected within a two‐week period with different temperature pairs set up at the same time and drawing from the same pool of individuals. Analyses of these crosses still find a difference between pair types in response to temperature ($\chi_{1}^{2}$ = 6.2, *p* = 0.013; *N* = 19), as does limiting the analysis to only pairs from January and March (*N* = 58, temperature by type interaction $\chi_{1}^{2}$ = 5.32, *p* = 0.02). Furthermore, comparing our March 23°C data to crosses between BV and HR collected in August 2019 for another study (*N* = 15; G. M. Kozak, unpublished data), we found no differences in the proportion that mated by the 3rd day (August = 56%, March = 62% Fisher's exact test, *p* =  1)or number of eggs laid by the third day (August = 3.06, March = 2.92, Mann–Whitney *U* test, *W* = 101.5, *p* = 0.93). This consistency of behavior in sampled individuals from HR and BV over an eight‐month period (~7 corn borer generations) suggests our findings are not due to differences in the subsamples of individuals included at different temperatures.

Our results suggest that elevated temperatures associated with climate change may increase reproductive investment in less preferred mates in European corn borers and potentially increase gene flow among populations. Any changes in gene flow could possibly facilitate the spread of adaptive genetic loci, such as those associated with resistance to Bt corn used in pest control and temperature tolerance which vary among strains (Coates & Siegfried, 2015; Wadsworth et al., 2020). Given that introgression of alleles among populations and species appears to be a major source of genetic novelty (Jones et al., 2018; Selz & Seehausen, 2019) that can aid in invasive species spread (Valencia‐Montoya et al., 2020), additional work is needed to explore pre‐ and postcopulatory temperature effects on mating between populations in insect pest and pollinator species.

## CONFLICT OF INTEREST

The authors declare that they have no conflicts of interest.

## AUTHOR CONTRIBUTION

**Arielle N. Enos:** Conceptualization (equal); Data curation (equal); Formal analysis (supporting); Methodology (equal); Writing‐original draft (supporting); Writing‐review & editing (equal). **Genevieve M. Kozak:** Conceptualization (equal); Data curation (equal); Formal analysis (lead); Funding acquisition (lead); Methodology (equal); Supervision (lead); Writing‐original draft (lead); Writing‐review & editing (equal).

## Supporting information

Figure S1‐S2

Table S1‐S2

## Data Availability

Data deposited in DRYAD: https://doi.org/10.5061/dryad.s7h44j172
