# Supplementary material for: Elevated temperature increases reproductive investment in less preferred mates in the invasive European corn borer moth
Source: Ecol Evol. 2021 Aug 4;11(17):12064–74. doi: 10.1002/ece3.7972 (PMC8427566; doi:10.1002/ece3.7972)
Supplement: Supplementary file 1 — Figure S1‐S2 [file ECE3-11-12064-s001.pdf]

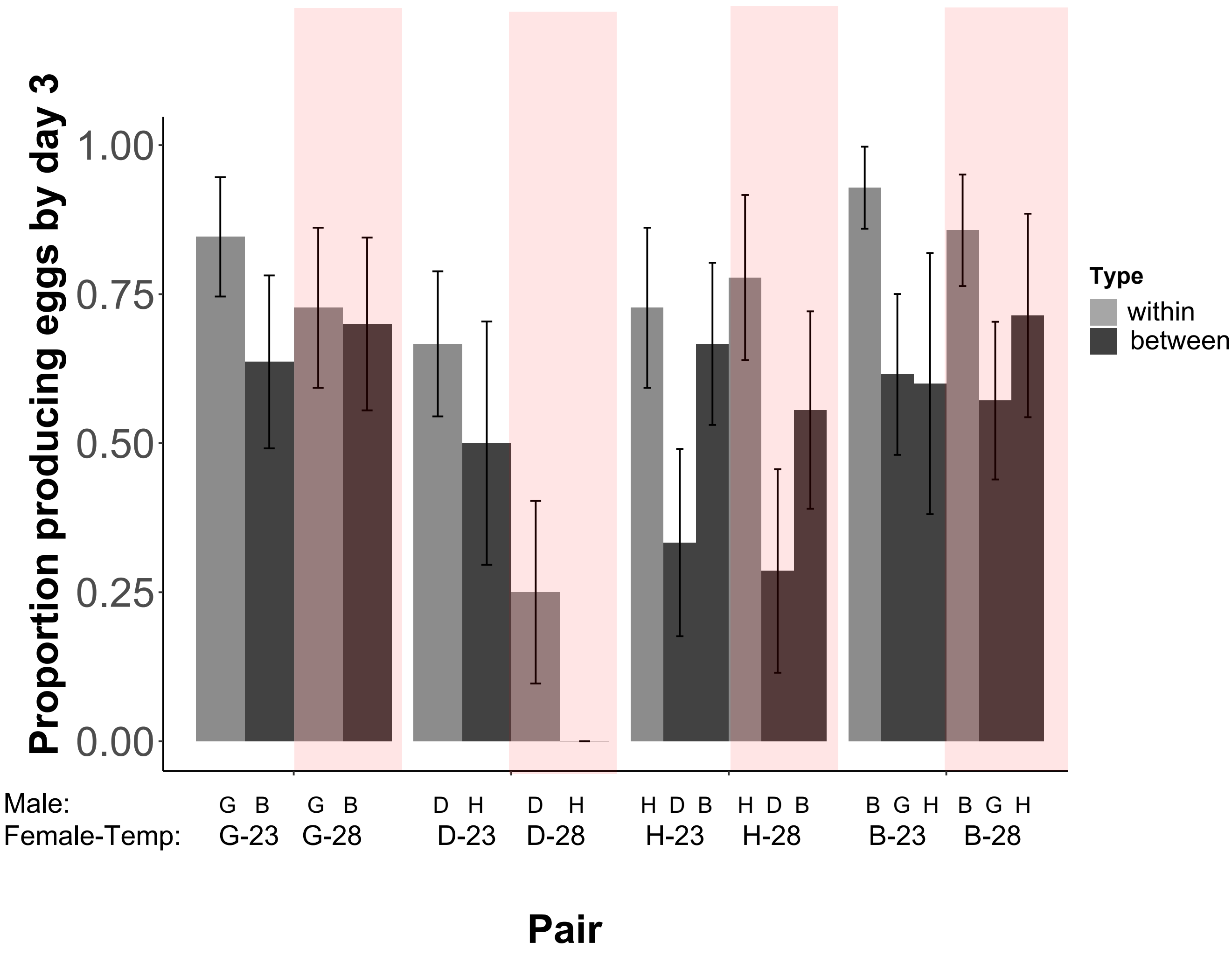

Appendix Figure 1. Proportion of pairs producing eggs by male line, female line, and temperature. Male line listed above, female line and temperature listed below. DV females were less likely to produce eggs than BV females at high temperature. Bars represent standard error of the proportion. Light gray = within-line pairs, dark grey between lines = between-line pairs, 28°C pairs shaded in red. No interactions between female line and temperature were significant. G = GE/Geneva D = DV/Dover; H = HR/Hurley, B = BV/Bouckville.

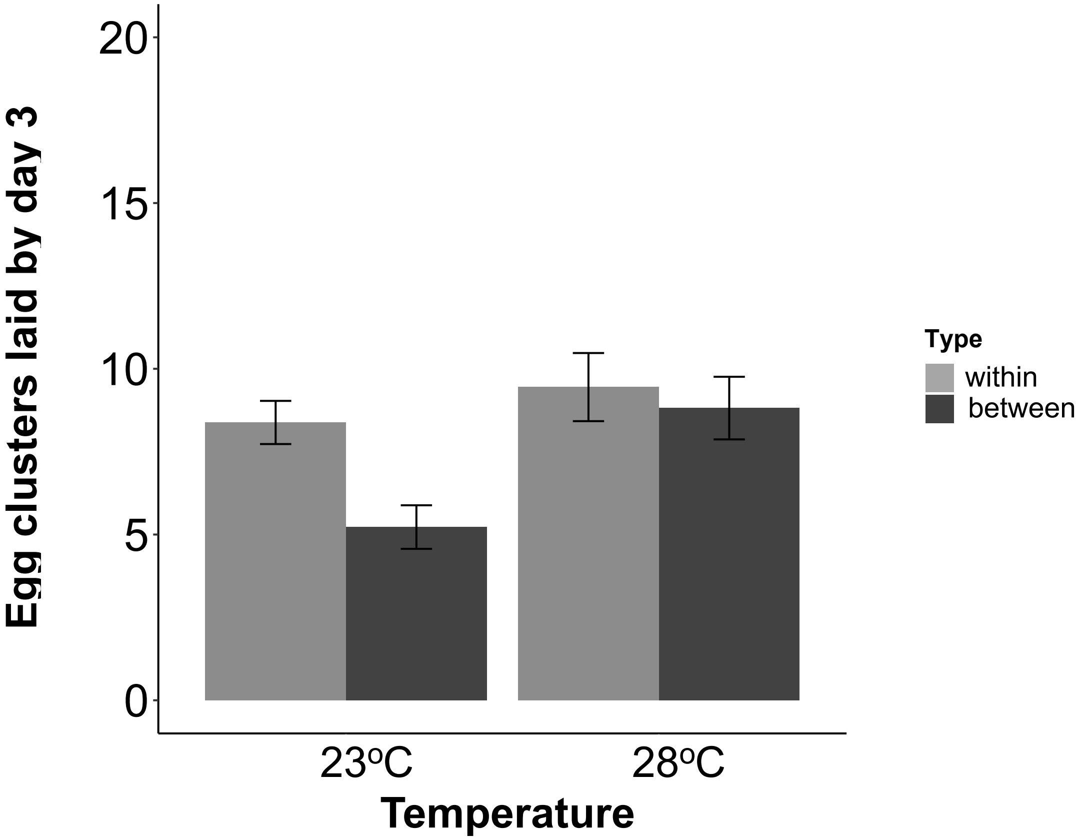

Appendix Figure 2. Mean eggs clusters laid by day 3 in within and between pairs raised at constant 23°C. Light gray= within-line pairs, dark grey = between-line pairs. Ambient within low =42, between N = 31; elevated within N =29, elevated between N = 27.
