# Supplementary material for: Elevated temperature increases reproductive investment in less preferred mates in the invasive European corn borer moth
Source: Ecol Evol. 2021 Aug 4;11(17):12064–74. doi: 10.1002/ece3.7972 (PMC8427566; doi:10.1002/ece3.7972)
Supplement: Supplementary file 2 — Table S1‐S2 [file ECE3-11-12064-s002.docx]

**APPENDIX**

**Appendix Table 1. Larval temperatures experienced by crosses**

| **Cross**  **(Female x Male)** | **Totalpairs**  **23**ºC | **Total Pairs**  **28**ºC | **Pairs**  **23**ºC  **23**ºC | **Pairs**  **28**ºC  **23**ºC | **Pairs**  **23**ºC  **26**ºC | **Pairs**  **28**ºC  **26**ºC | **Pairs**  **23**ºC  **28**ºC*  1 week | **Pairs**  **28**ºC  **28**ºC*  1 week |
| --- | --- | --- | --- | --- | --- | --- | --- | --- |
| BV x BV | 14 | 14 | 9 | 11 | 5 | 3 | 0 | 0 |
| DV x DV | 15 | 8 | 15 | 8 | 0 | 0 | 0 | 0 |
| HR x HR | 11 | 9 | 11 | 9 | 0 | 0 | 0 | 0 |
| GE x GE | 11 | 13 | 11 | 3 | 0 | 8 | 0 | 0 |
| HR x DV | 9 | 7 | 9 | 7 | 0 | 0 | 0 | 0 |
| DV x HR | 6 | 1 | 6 | 1 | 0 | 0 | 0 | 0 |
| HR x BV | 10 | 9 | 9 | 9 | 1 | 0 | 0 | 0 |
| BV x HR | 5 | 7 | 5 | 7 | 0 | 0 | 0 | 0 |
| GE x BV | 11 | 10 | 0 | 4 | 6 | 6 | 5 | 0 |
| BVx GE | 13 | 14 | 0 | 1 | 8 | 13 | 5 | 0 |

**Appendix Table 2. Number that laid vs. did not lay egg clusters by day 3**

|  | **Pairs**  **23**ºC | | **Pairs**  **28**ºC | |
| --- | --- | --- | --- | --- |
| **Cross**  **(Female x Male)** | Laid eggs | No eggs | Laid eggs | No eggs |
| BV x BV | 13 | 1 | 12 | 2 |
| DV x DV | 10 | 5 | 2 | 6 |
| HR x HR | 8 | 3 | 7 | 2 |
| GE x GE | 11 | 2 | 8 | 3 |
| HR x DV | 3 | 6 | 2 | 5 |
| DV x HR | 3 | 3 | 0 | 1 |
| HR x BV | 7 | 3 | 5 | 4 |
| BV x HR | 3 | 2 | 5 | 2 |
| GE x BV | 7 | 4 | 7 | 3 |
| BV x GE | 8 | 5 | 8 | 6 |
| **Total** | **73** | **33** | **56** | **33** |
